# Total esophagogastric dissociation (TEGD) in neurologically impaired children: the floor to parents

**DOI:** 10.1007/s13304-022-01384-5

**Published:** 2022-09-21

**Authors:** Giovanni Parente, Sara Maria Cravano, Marco Di Mitri, Eduje Thomas, Chiara Girella, Simone D’Antonio, Tommaso Gargano, Mario Lima

**Affiliations:** Pediatric Surgery Department, IRCCS Sant’Orsola University Hospital, Alma Mater Studiorum–University of Bologna (IT), via Massarenti 9, 40138 Bologna, Italy

**Keywords:** Total esophagogastric dissociation, TEGD, Neurologically impaired children, GERD, Fundoplication, QoL, Abdominal surgery

## Abstract

Total esophagogastric dissociation (TEGD) was proposed to treat gastroesophageal reflux disease (GERD) both as a rescue in case of fundoplication failure and as first-line surgery in neurologically impaired children (NIC). Aim of the study is to evaluate the impact of TEGD on the quality of life (QoL) of both NIC and their caregivers focusing on the parents’ point of view. A retrospective observational study was conducted on all NIC who underwent TEGD in our center between 2012 and 2022. A questionnaire centered on the parents’ point of view and investigating QoL of NIC and their caregivers was administered to all patients’ parents. Data were compared using Fisher exact test and Mann–Whitney test; a *p*-value < 0.05 was considered statistically significant. 12 patients were enrolled in the study. Parents reported improvements in weight gain (*p* = 0.03), sleep disorders, apnea, regurgitation and vomiting (*p* < 0.01). Caregivers also declared a decrease in number of hospitalizations, particularly related to severe respiratory infections and *ab ingestis* pneumonia (*p* = 0.01). We also documented a reduction of caregivers’ worries during food administration (*p* < 0.01). 50% of parents whose children were subjected to both fundoplication and TEGD would suggest TEGD as first line surgical treatment instead of fundoplication. According to parents’ point of view, TEGD improves significantly NIC QoL and 50% of them would enthusiastically suggest TEGD as first-line surgical approach to GERD in NIC.

## Introduction

Gastroesophageal reflux disease (GERD) is a common affection interesting up to 70% of neurologically impaired children (NIC) causing serious malnourishment, vomiting, aspiration pneumonia and sleep disorders [1]. A fundamental difference between healthy children and NIC is that, in the latter, the natural history of this disease rarely evolves toward resolution, due to persistence or even worsening of factors such as esophageal dysmotility, delayed gastric emptying, poor posture, repeated seizures, scoliosis and drugs that may favorite gastroesophageal reflux. Treatment of GERD in these patients represents a challenge since conservative management is often not sufficient. Although surgical treatments such as Nissen fundoplication must be considered, they have poor results in NIC, with even higher probability of failure in case of redo [2–4].

In 1997, Adrian Bianchi described for the first time a new surgical approach named total esophagogastric dissociation (TEGD).

Thought initially as a last choice, some authors have supposed that it might have already caught momentum for the future of GERD surgery [5]. In the last 20 years, major technical progresses have been made and now TEGD is considered feasible even with minimally invasive approaches.

Having established feasibility and efficacy of TEGD, surgeons should deal with its impact on QoL not only of NIC themselves but even of their caregivers [6]. NIC’s parents must spend a lot of time and energies to cope with the needs of this fragile population. Feeding NIC is time consuming and requires long lasting meals to avoid GER as well as being a threat considering the high rates of aspiration and apnea. Therefore, TEGD not only improves NIC clinical conditions and consequently their QoL but can even reduce caregivers’ stress and anxiety with positive repercussions on the entire family [7].

Aim of the study is to investigate mid- and long-term clinical outcome of TEGD according to parents’ point of view and to collect the latter impressions on the improvements in families’ everyday life.

## Methods

### Study design and population

A retrospective observational study was conducted in our Department of Pediatric Surgery, IRCCS Sant’Orsola-Malpighi University Hospital of Bologna, following Ethical Committee approval (CHPED-21-02-DEG). Clinical records were retrospectively analyzed to find out all neurologically impaired children who underwent TEGD for GERD in our department between January 2011 and January 2021. Patients with less than one-year follow-up were excluded from the study.

**Fig. 1:**
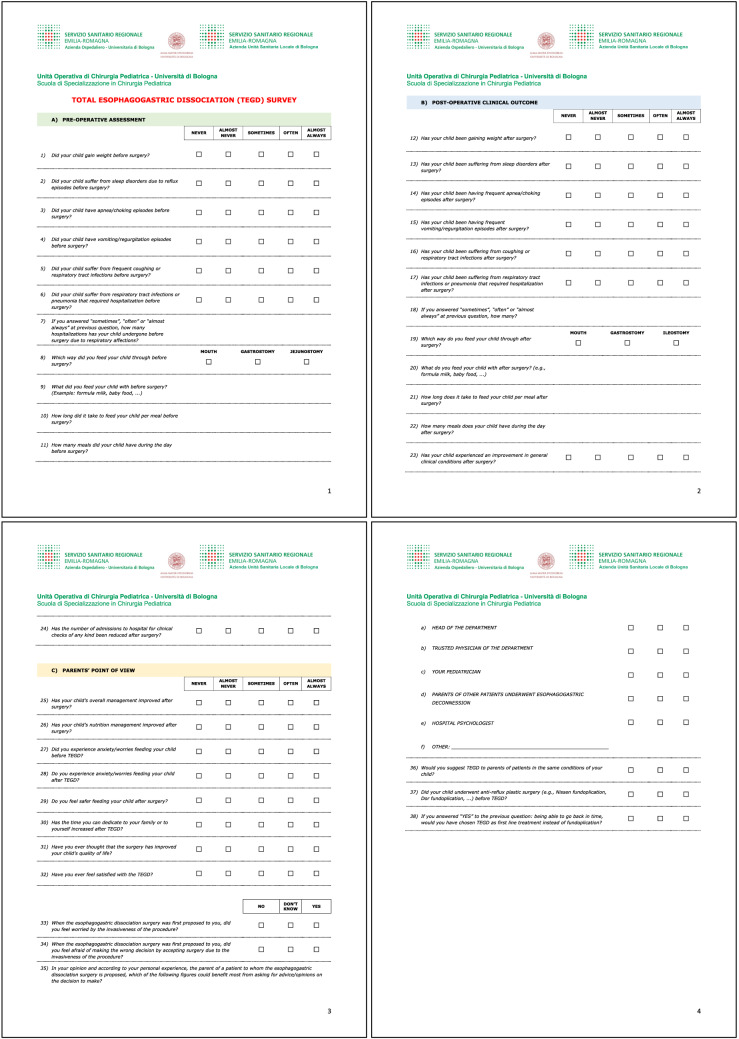
Questionnaire administered to patients' caregivers

A questionnaire (Fig. 1) was administered to all patients enrolled in the study investigating:

- Pre-operative data, including weight gain, sleep disorders, apnea, vomit, cough, episodes of hospitalization due to airways infections, time required for feeding in terms of number and duration of meals.
- Post-operative data: the same parameters asked for the pre-operative time were investigated to compare results.
- Parents’ point of view on clinical outcome, improvement on feeding management, anxiety during food administration, QoL and satisfaction after TEGD.

For each question, parents could give answer with a score from 1 to 5: 1 = never, 2 = almost never, 3 = sometimes, 4 = often, 5 = almost always.

For 6 questions in the parents’ point of view section, caregivers could answer “yes”, “no” or “I don’t know”.

### Surgical procedure

Indication to TEGD is failure of fundoplication with recurrence of GER and/or recurrent aspiration due to significant swallowing discoordination.

**Fig. 2:**
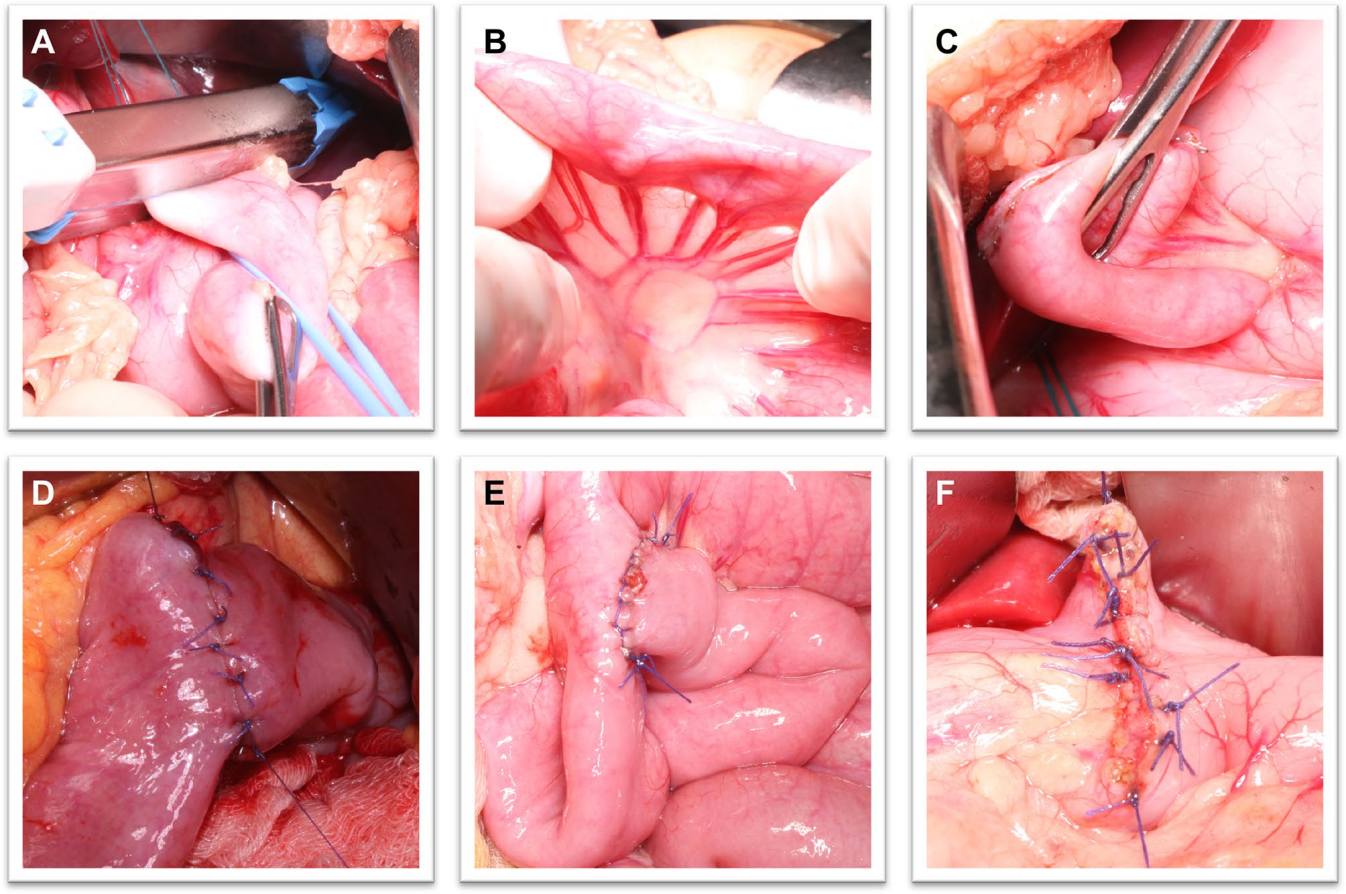
Open TEGD (for details, please read the "surgical procedure" section of methods)

TEGD, in our center, is performed with open approach. This surgical technique features a Roux-en-Y esophagojejunal anastomosis and a jejunal end-to-side anastomosis with a gastrostomy opening. Access to the peritoneal cavity is obtained through a longitudinal median xipho-pubic incision. The abdominal esophagus is isolated at the esophageal hiatus and detached from the stomach with a linear stapler (Fig. 2A). The jejunum is then examined and sectioned 20–30 cm distally to the ligament of Treitz (Fig. 2B). The distal stump is brought up to perform an end-to-side anastomosis with the esophageal stump (Fig. 2C, D), whereas the proximal jejunal stump is mobilized for a Roux-en-Y jejunum-jejunal end-to-side anastomosis (Fig. 2E). Being the exclusion of the stomach pivotal to achieve a permanent resolution of gastroesophageal reflux, it is necessary to fashion a gastrostomy for feeding purposes. On the other hand, the esophagojejunal anastomosis ensures the swallowing of saliva and small meals. The last step of the procedure entails a pyloroplasty to prevent gastroparesis, since the vagus nerves are often severed during the esophagogastric dissociation (Fig. 1F) [8].

### Statistical analysis

Continuous data are presented as mean ± standard deviation (DS) and median. Data were analyzed with Fisher exact test and Mann–Whitney test. A *p*-value < 0.05 was considered statistically significant.

## Results

Between January 2011 and January 2021, 15 NIC, 8 (53.3%) females and 7 (46.7%) males, underwent TEGD for GERD at our Pediatric Surgery department. Mean age at surgery was 7.9 ± 8.1 years old (range: 1–27 y.o.).

Causes of neurologic impairment were: 7 (46.0%) cases of hypoxic-ischemic encephalopathy (HIE), 1 (13.3%) PIGN gene mutation, 1 (6.7%) Moebius Syndrome, 1 (6.7%) CHARGE Syndrome, 1 (6.7%) meningitis sequelae, 1 (6.7%) pachygyria, 1 (6.7%) Ellis Van Creveled Syndrome, 1 (6.7%) Trisomy 18. Data are resumed in Table 1

**Table 1:** Causes of neurologic impairment in patients who underwent TEGD

Causes of neurologic impairment	No. (%)
Hypoxic-ischemic encephalopathy	7 (46.0%)
PIGN gene mutation	2 (13.3%)
Moebius Syndrome	1 (6.7%)
CHARGE Syndrome	1 (6.7%)
Tetraparesis due to meningitis	1 (6.7%)
Pachygyria	1 (6.7%)
Ellis Van Creveled Syndrome	1 (6.7%)
Trisomy 18	1 (6.7%)

7 (46.7%) patients were subjected to gastrostomy opening and 4 (26.7%) underwent Nissen fundoplication before TEGD, while 4 (26.7%) were subjected directly to TEGD.

3 (20%) patients passed many years after surgery due to respiratory failure because of worsening of their diseases; therefore, 12 (80%) were enrolled in the study.

Parents reported a statistically significant improvement in terms of weight gain during post-operative follow-up (*p* = 0.02), reduction of sleep disorders and apnea episodes (*p* < 0.01).

**Fig. 3:**
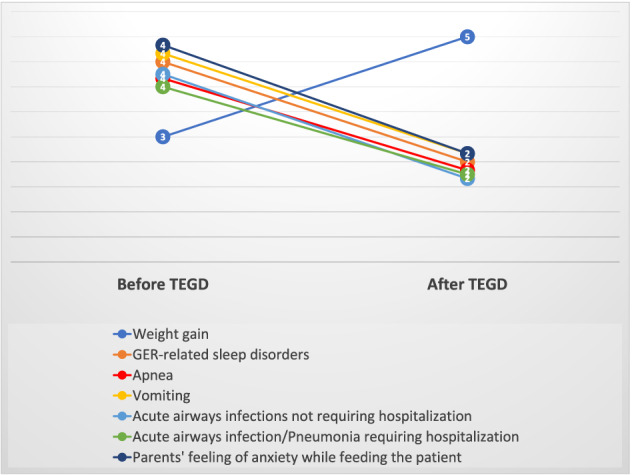
Results of TEGD on the main outcomes investigated. Chart shows the median of the answers before and after surgery (1 = never, 2 = almost never, 3 = sometimes, 4 = often, 5 = almost always)

Vomit and regurgitate decreased (*p* < 0.01) as well as respiratory symptoms like cough, airways infections (*p* < 0.01) and hospitalizations for pneumonia (*p* = 0.01). Data are showed in Fig. 3.

**Fig. 4:**
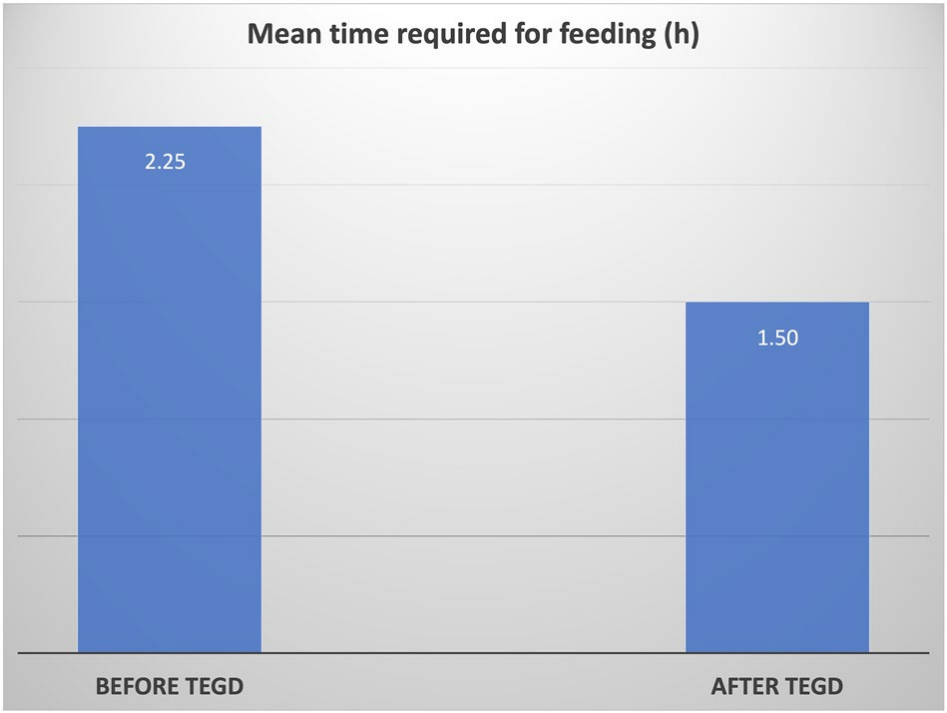
Mean time required for feeding before and after TEGD

Caregivers seemed more confident and less scared during food administration after TEGD (*p* < 0.01), and the time required to feed the patients drastically decreased from a mean of 4.9 ± 5.8 h (range: 0.25–17 h) to 2.4 ± 3.1 h (range: 0.8–12 h) per meal (Fig. 4).

Between the 4 patients who underwent both Nissen fundoplication and TEGD, caregivers said in 2 cases (50%) that would have preferred TEGD as first-line treatment, while the other 2 (50%) still agree with the decision of Nissen fundoplication and then TEGD in case of failure.

## Discussion

Gastroesophageal reflux disease is particularly common in NIC with an incidence up to 70% [9, 10].

GERD can arise with plenty of symptoms such as regurgitation, irritability, emesis, feeding refusal, growth retardation, epigastric pain, chronic cough, hoarseness, halitosis, dental erosions, apnea and *ab ingestis* pneumonia [11–13]. These conditions make NIC’s care complex especially for parents who often spend most of their time and energies to cope with their children needs [14].

To demonstrate how GERD can heavily affect everyday life of children, Marlais et all, in a retrospective observational study, investigated QoL in 40 children between 5 and 18 years old, who presented with gastrointestinal symptoms at their center from February to May 2009. They showed how children with GERD had a significantly lower QoL score than children with IBD (74.0 vs. 81.8, *p* < 0.01) and healthy children (74.0 vs. 84.4, *p* < 0.01) [15].

Various possibilities are available to diagnose gastroesophageal reflux (GER). Commonly, 24 h pH-impedancemetry is used to evaluate GER, because it measures both the pH value and the retrograde or anterograde bolus transport in the esophagus, thereby allowing the detection of all episodes of reflux over a 24-h period [16].

An upper gastrointestinal barium contrast study is mainly helpful to detect anatomical reasons of GER and to assess its severity.

GERD unresponsive to conservative treatment is a challenge for pediatricians and pediatric surgeons. There are several surgical techniques to increase pressure on the lower esophageal sphincter (LES) to avoid reflux in pediatric age such as Nissen, Dor, Toupet and Thal fundoplication. Nowadays, the gold standard technique to treat reflux in NIC is considered laparoscopic or robotic Nissen fundoplication [17]. When minimally invasive approaches are not advisable, open technique is necessary [18].

If reflux persists, a redo surgery is reasonable but the feasibility of a laparoscopic approach decreases from 89 to 68% in case of a second revision [19, 20].

In 1997 Adrian Bianchi suggested for the first time TEGD in NIC when fundoplication did not show clinical improvement or as a first-line alternative approach in GERD [5, 21].

Lall et al. analyzed their experience with 50 patients who underwent TEGD: 34 as primary approach and 16 as rescue after failure of fundoplication. They state that TEGD is safe, feasible, with low post-operative mortality and morbidity suggesting it as first-line surgical treatment in severe neurologic impairment with GERD coupled to significant oropharyngeal incoordination [22].

Coletta et al. too, analyzing 66 NIC who underwent TEGD for GERD, showed how it seemed a reliable surgical option as both primary or rescue procedure [23].

Buratti et al. comparing Nissen fundoplication with TEGD concluded that the latter guarantees best results in terms of improvements of all anthropometric and nearly all biochemical parameters taken in exam and the decrease of episodes of respiratory infections, hospitalizations and feeding time [6, 24, 25].

More than 20 years after the first proposal, considering also the experiences reported in literature and discussed above, we can definitely assume that, when performed by experienced surgeons, TEGD is a safe, feasible and effective procedure. Thus, it may be redundant to discuss about surgical details or outcome of this procedure actually [26, 27].

What we think should be better investigated is the point of view of caregivers in terms not only of efficacy of TEGD but also how it can impact the everyday family life if patients.

In our study, parents reported improvements in weight gain, a decrease in episodes of post-prandial regurgitation and vomit and apnea, better sleep quality, less need of hospitalization especially for *ab ingestis* pneumonia [28].

According to aim of the study, the data that interested us the most were the following. Parents declared to fell less frustrated and scared when feeding their children. This extremely important result is a consequence of the improvement of GER symptoms that made nutrition easier and safer.

Moreover, parents declared a decrease of the time required to administrate a single meal to their children.

If we couple the two previous findings with the reduction of hospitalizations, it is clear how this surgical procedure affected positively the QoL of caregivers and their family: parents are less frustrated and can spend and enjoy more time with their children doing activities other than simply feeding them and taking care of their medical needs.

Moreover, happier parents, better NIC’s general conditions, less time spent in hospital and more free time improve the whole family daily life quality.

In our cohort, 4 patients underwent a Nissen fundoplication, which proved ineffective in preventing GER, prior to being subjected to TEGD. Upon completion of the surgical iter, the parents of two of these patients (50%) admitted they would have preferred TEGD at first instance. Such decision is entirely acceptable and deserves full support based on the consideration that quality of life of NIC is profoundly hampered by the underlying conditions and GER. Therefore, parents are often exhausted and request a single definitive solution. Moreover, as experience with this procedure increases, there is a growing number of authors proposing TEGD as a primary surgical treatment in NIC, rather than a rescue procedure.

On the other hand, we fully understand the reasons of the remaining two parents (50%), who declared to agree with a first attempt with the fundoplication before proceeding to TEGD. TEGD is a major surgical procedure which implies a total disruption of the anatomy of the child, making it difficult to be welcomed initially.

The present study, at our knowledge, is the only one in literature entirely focused on parents’ perception of the TEGD’s outcome. Although, it harbors some limitations.

First, a study that uses a not validated questionnaire may be subject to measurement error and the conclusions drawn cannot be made with total confidence. On the other hand, there is not a validated survey for this specific population and the common PedsQL questionnaires cannot be applied to NIC.

Moreover, the low numerosity impedes us from generalizing our conclusions. Nevertheless, we are convinced that our results, of extreme interest for pediatric surgeons, could encourage other centers to investigate the topics faced in this paper with further multicentric studies.

## Conclusion

Parents of NIC who underwent TEGD are enthusiastic of the outcome obtained reporting improvements in almost all the areas investigated (symptoms, quality of sleep, respiratory infections and *ab ingestis* pneumonia, weight gain, general conditions).

Moreover, the reduction of GER symptoms during feeding made this procedure safer and easier and parents feel less scared and stressed during this important activity.

Feeding duration after TEGD decreased and that gives caregivers the possibility to have more time that can be spent in other activities with their child and the rest of the family. This is even more true considering the reduction of episodes of hospitalization reported after TEGD.

50% of caregivers of NIC who underwent Nissen fundoplication and, due to failure, subsequent TEGD, would choose TEGD as first-line treatment for GERD.

Further studies are needed to confirm our results and to discuss the opportunity of TEGD as first-line treatment for GERD in NIC.
